# Assessment of Infantile Mineral Imbalances in Autism Spectrum Disorders (ASDs)

**DOI:** 10.3390/ijerph10116027

**Published:** 2013-11-11

**Authors:** Hiroshi Yasuda, Toyoharu Tsutsui

**Affiliations:** La Belle Vie Research Laboratory, 8-4 Nihonbashi-Tomizawacho, Chuo-ku, Tokyo 103-0006, Japan

**Keywords:** autism spectrum disorders, etiology of neurodevelopment disorders, infantile zinc deficiency, toxic metal burdens, metallomics profiles, epigenetic alterations, infantile window

## Abstract

The interactions between genes and the environment are now regarded as the most probable explanation for autism. In this review, we summarize the results of a metallomics study in which scalp hair concentrations of 26 trace elements were examined for 1,967 autistic children (1,553 males and 414 females aged 0–15 years-old), and discuss recent advances in our understanding of epigenetic roles of infantile mineral imbalances in the pathogenesis of autism. In the 1,967 subjects, 584 (29.7%) and 347 (17.6%) were found deficient in zinc and magnesium, respectively, and the incidence rate of zinc deficiency was estimated at 43.5% in male and 52.5% in female infantile subjects aged 0–3 years-old. In contrast, 339 (17.2%), 168 (8.5%) and 94 (4.8%) individuals were found to suffer from high burdens of aluminum, cadmium and lead, respectively, and 2.8% or less from mercury and arsenic. High toxic metal burdens were more frequently observed in the infants aged 0–3 years-old, whose incidence rates were 20.6%, 12.1%, 7.5%, 3.2% and 2.3% for aluminum, cadmium, lead, arsenic and mercury, respectively. These findings suggest that infantile zinc- and magnesium-deficiency and/or toxic metal burdens may be critical and induce epigenetic alterations in the genes and genetic regulation mechanisms of neurodevelopment in the autistic children, and demonstrate that a time factor “infantile window” is also critical for neurodevelopment and probably for therapy. Thus, early metallomics analysis may lead to early screening/estimation and treatment/prevention for the autistic neurodevelopment disorders.

## 1. Introduction

ASDs are a group of neural development disorders characterized by impairments in social interaction and communication, and by the presence of restricted and repetitive behaviours [1,2]. Clarification of the pathogenesis and effective treatment of autism spectrum disorders (ASDs) is one of the challenges today. ASDs continue to increase in prevalence up to 1 in 88 children [1,2,3] and are known to be highly heritable (~90%), and some related genes have been reported [4,5,6,7,8]. However, the underlying genetic determinants are still not clarified [1,9], and the interaction of heritable factors with uncertified lifestyle and environmental factors seem play a significant role in the pathogenesis. For example, organic mercury had been claimed one of environmental candidates causing autistic disorders [10,11,12], but its relationship remains to be established. Recently, epigenetic alteration of gene expression by environmental factors is considered one of key events in the pathogenesis of genetic diseases [13,14], and some toxic elements such as cadmium and arsenic have been reported to be candidate factors that induce epigenetic alterations [15,16,17,18,19] and neurodevelopmental disorders [20].

Recent great advances in high-sensitive and reliable trace element analysis method using inductively coupled plasma mass spectrometry (ICP-MS) have enabled it to be applied for forensic medical research and estimating chronic toxic metal burden and mineral deficiency in the human body [21,22]. Thus, the clinical application of reliable hair mineral analysis methods based on ICP-MS has been tried to investigate the association of some diseases/symptoms with trace bio-element kinetics including toxic metals and essential minerals [23,24,25,26,27,28].

For the last seven years, we have examined the association of toxic metal burdens with autistic disorders, and reported that some of the autistic children have suffered from high accumulation of toxic metals such as cadmium, lead or aluminium [29,30,31], and recently demonstrating the association with infantile zinc deficiency [32,33].

In this overview article in which human scalp hair concentrations of 26 trace elements have been examined for 1,967 children with autistic disorders aged 0–15 years, we demonstrate that many of the patients, especially in the infants aged 0–3 years-old, are suffering from marginal to severe zinc- and magnesium-deficiency and/or high burdens of several toxic metals such as aluminium, cadmium and lead, indicating the presence of a critical term “infantile window” in neurodevelopment and probably for therapy.

## 2. Mineral Disorders in Autism

### 2.1. Infantile Zinc Deficiency

**Figure 1:**
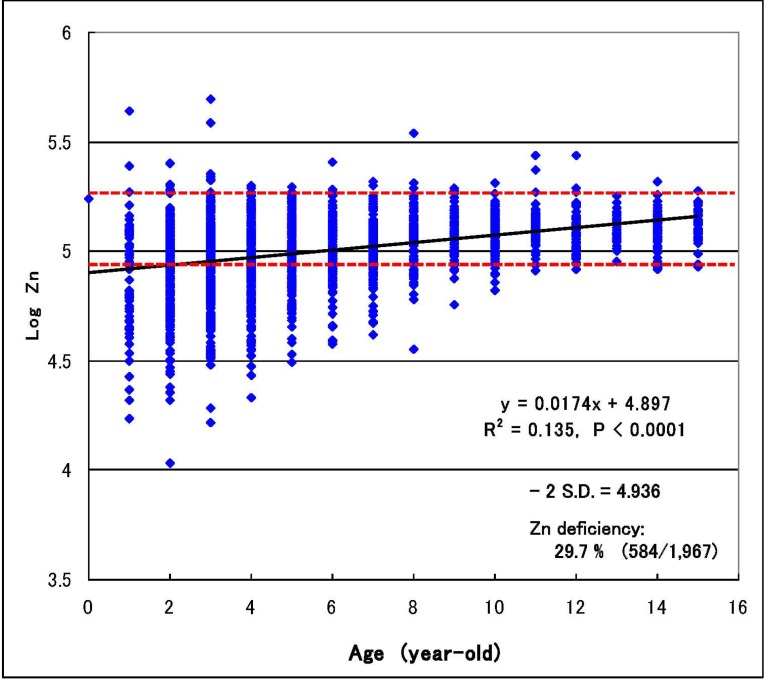
Relation of logarithmic zinc concentration with age in autistic children [33].

The histogram of hair logarithmic zinc concentrations for 1,967 autistic children diagnosed by their physicians was non-symmetric with tailing in lower range, and 584 in 1,967 subjects (29.7%) were found to have a lower zinc concentration than—2 S.D. (standard deviation) level of the reference range (86.3–193 ppm; geometric mean = 129 ppm), estimated as zinc deficiency. The incidence rates of zinc deficiency in the age groups of 0–3, 4–9 and 10–15 years-old were estimated 43.5%, 28.1% and 3.3% in male and 52.5%, 28.7% and 3.5% in female, and a significant correlation of zinc concentration with age (*r* = 0.367, *p* < 0.0001) was observed (Figure 1), suggesting that infants are more liable to zinc deficiency than elder children. The minimum zinc concentration of 10.7 ppm was detected in a 2-year-old boy, corresponding to about 1/12 of the mean reference level. The zinc concentration of only one 0-year-old case (11 months-old) was 173 ppm in the normal range and seem to be a suspected case, because she was suffered from high burdens of aluminium (52.5 ppm), lead (9.1 ppm), iron (12.8 ppm) and copper (134 ppm). There was little marked gender difference in hair zinc concentration and incidence rate of zinc deficiency.

The association of hair logarithmic zinc concentration with age in autistic children (N = 1,967) is shown. Each point represents the corresponding age and logarithmic zinc concentration (ng/g hair: ppb) of the individual child. The dotted horizontal lines represent the ±2 S.D. levels of the reference range (86.3–193 ppm) of hair zinc concentrations. A significant relation of the zinc concentration with age (*r* = 0.367, *p* < 0.0001) in the autistic children is shown [33].

### 2.2. Infantile Magnesium/Calcium Deficiency

Following to zinc deficiency, magnesium and calcium deficiency was observed in 347 (17.6%) and 114 (5.8%) individuals in the autistic children, and for the other essential metals such as iron, chromium, manganese, copper and cobalt, their incidence rates of deficiency were 2.0% or less (Table 1). The incidence rates of magnesium deficiency in the age groups of 0–3, 4–9 and 10–15 years-old were 27.0%, 17.1% and 4.2% in male and 22.9%, 12.7% and 4.3% in female subjects, and a significant correlation of magnesium concentration with age (*r* = 0.362, *p* < 0.0001) was observed, suggesting that infants are also liable to magnesium deficiency than elder children. The minimal magnesium concentration of 3.88 ppm was detected in a 2-year-old girl, corresponding to almost 1/10 of the mean reference level (39.5 ppm). Considerable calcium deficiency rate was observed only in lower age groups less than 10 years-old.

**Table 1:** Prevalence of mineral deficiency in autistic children [33].

Mineral	Number of Cases with Deficiency	Rate (%) of Deficiency
Zn	584	29.7
Mg	347	17.6
Ca	114	5.8
Co	40	2.0
Fe	17	0.9
Cr	12	0.6
Mn	4	0.2
Cu	4	0.2

The number and incidence rate of individuals with mineral deficiency (lower than −2 S.D.) in 1,967 autistic children (1,553 males and 414 females) are shown in the table [33].

### 2.3. Toxic Metal Burdens

In contrast to essential metals, high body burdens of some toxic metals such as aluminium, cadmium and lead of over their +2 S.D. levels were observed in 339 (17.2%), 168 (8.5%) and 94 (4.8%) individuals, respectively, and their incidence rates of high burden were higher than that of mercury and arsenic (2.8% and 2.6%) (Table 2). 

**Table 2:** Prevalence of high toxic metal burden and the maximum level in autistic children [33].

Toxic Metal	Number of Cases with High Burden	Rate (%) of High Burden	Maximum (ppm)	Ratio to Reference
Al	339	17.2	79.4	21.1
Cd	168	8.5	5.5	782.0
Pb	94	4.8	24.9	57.4
Hg	56	2.8	36.3	9.3
As	52	2.6	1.7	33.5

The number and incidence rate of individuals with high toxic metal burden (higher than +2 S.D.) in 1,967 autistic children (1,553 males and 414 females) and the maximum concentration are tabled [33].

High toxic metal burdens were more frequently observed in the infants aged 0–3 years-old: that is, the incidence rate was 20.6%, 12.1%, 7.5%, 3.2% and 2.3% for aluminium, cadmium, lead, arsenic and mercury. The detected maximal concentration of aluminium, cadmium, lead, mercury and arsenic was 79.4 ppm, 5.47 ppm, 24.9 ppm, 36.3 ppm and 1.7 ppm, respectively, corresponding to 21-, 782-, 57-, 9- and 33-fold of each mean reference level.

**Figure 2:**
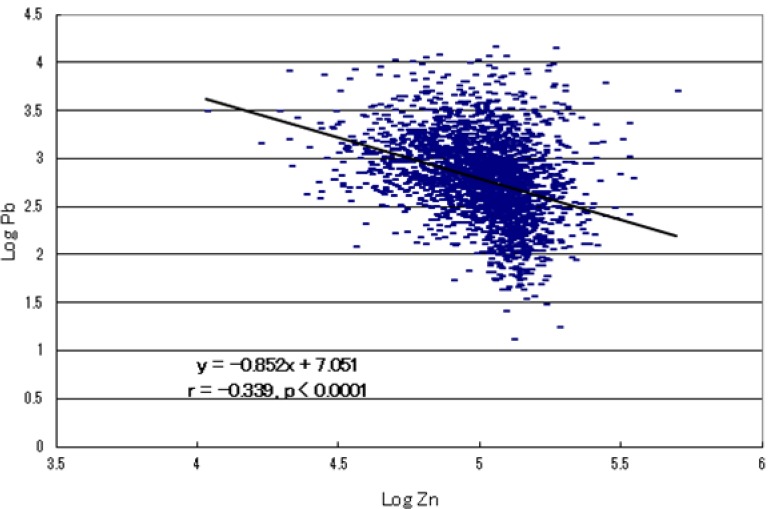
Inverse relation of zinc and lead concentration in autistic children.

A high significant inverse relationship between zinc and lead concentrations (*r* = −0.339, *p* < 0.0001; Figure 2), and also aluminium (*r* = −0.247) and cadmium (*r* = −0.198) concentrations, was observed, suggesting that these toxic metal burdens associate with infantile zinc deficiency.

The inverse relationship between hair logarithmic zinc and lead concentration in autistic children (N = 1,967) is shown. Each point represents the corresponding logarithmic zinc and lead concentration of the individual child. A high significant inverse relationship between zinc and lead concentrations (*r* = −0.339, *p* < 0.0001) in the autistic children is shown.

### 2.4. Metallomics Profiles in Autistic Infants

**Figure 3:**
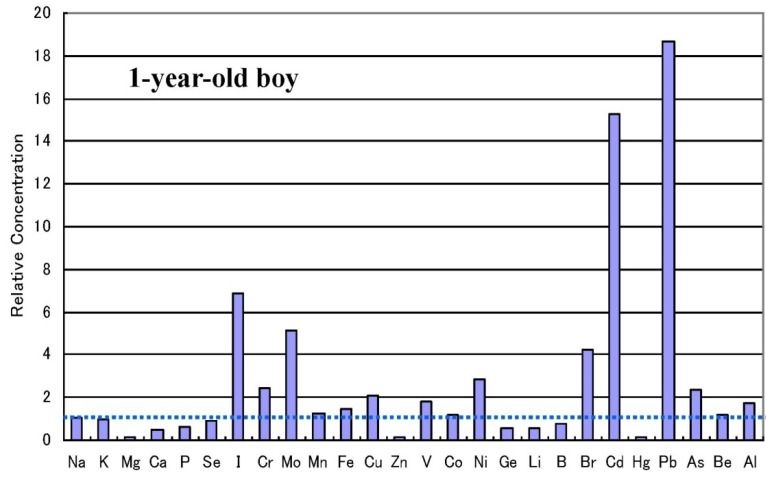
Metallomics profile of an autistic child with high cadmium and lead burdens [33].

**Figure 4:**
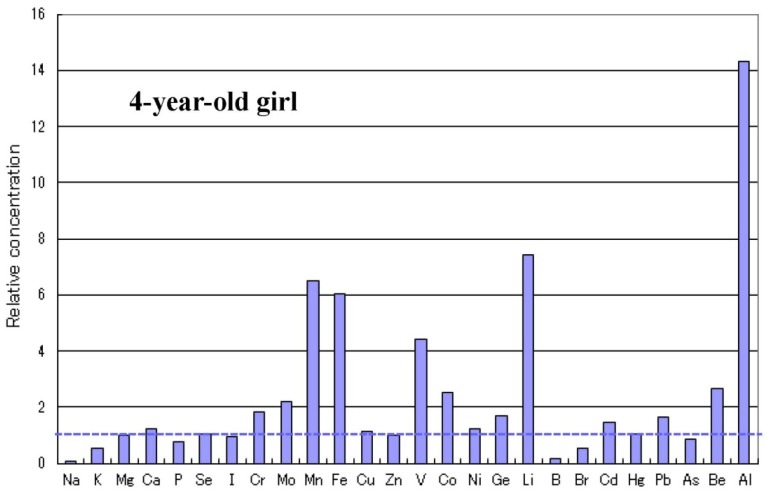
Metallomics profile of an autistic child with high aluminium burden.

**Figure 5:**
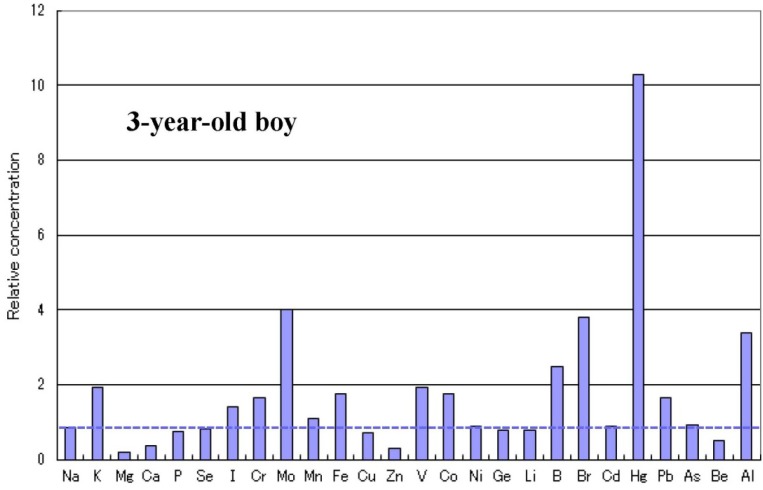
Metallomics profile of an autistic child with high mercury burden.

**Figure 6:**
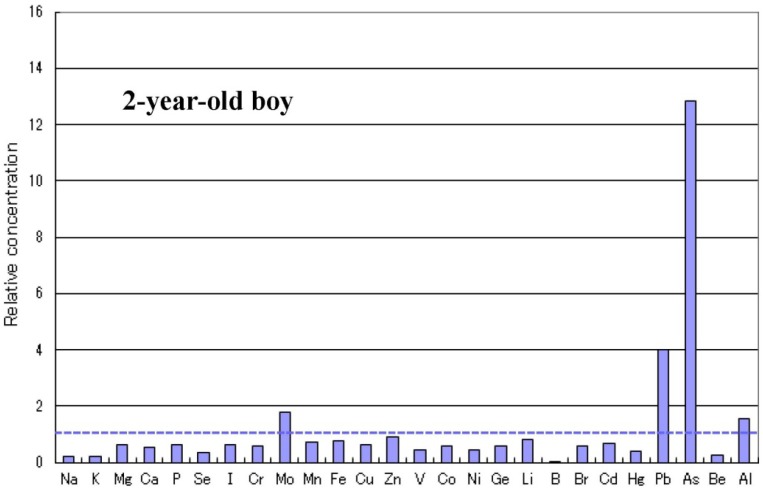
Metallomics profile of an autistic child with high arsenic burden.

**Figure 7:**
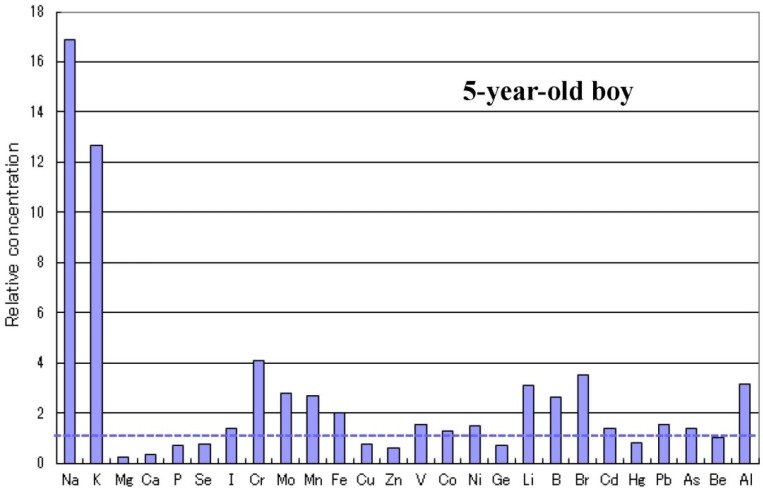
Metallome profile of an autistic child with high sodium and potassium levels.

There are some sub-types observed in the metallomics profiles characteristic in autistic children. Figure 3 shows a representative autistic profile in a 1-year-old boy suffering from severe zinc- and magnesium-deficiency and simultaneous high burdens of cadmium and lead. The other autistic metallomics profiles with high burdens of aluminium, mercury or arsenic are shown in Figure 4, Figure 5 and Figure 6. Figure 7 shows a unique profile with high sodium and potassium concentrations, a characteristic profile detectable in hair specimens. It remains to be clarified which type of metallomcs profiles corresponds to which type of autism spectrum disorders.

A representative metallomics profile of a 1-year-old boy with autism is shown, exhibiting severe zinc- and magnesium-deficiency and simultaneous high burdens with cadmium (107 ppb) and lead (8.11 ppm). Each bar represents the relative concentration of the respective trace element in his scalp hair specimen. The dotted horizontal line at 1.0 represents the reference control level of each trace element [33].

A representative metallomics profile of 4-year-old girl with autism is shown, exhibiting simultaneous high burdens with aluminium (53.7 ppm), manganese (702 ppb), iron (37.0 ppm) and lithium (12.2 ppb). Each bar represents the relative concentration of the respective trace element in her scalp hair specimen. The dotted horizontal line at 1.0 represents the reference control level of each trace element.

A metallomics profile of a 3-year-old boy with autism is shown, exhibiting a high burden with mercury of over 10-fold level (40.1 ppm). Each bar represents the relative concentration of the respective trace element in his scalp hair specimen. The dotted horizontal line at 1.0 represents the reference control level of each trace element.

A metallomics profile of a 2-year-old boy with autism is shown, exhibiting a high burden with arsenic of over 12-fold level (655 ppb). Each bar represents the relative concentration of the respective trace element in his scalp hair specimen. The dotted horizontal line at 1.0 represents the reference control level of each trace element.

A metallome profile of 5-year-old boy with autism is shown, exhibiting simultaneous high levels of sodium (335 ppm) and potassium (210 ppm). Each bar represents the relative concentration of the respective trace element in his scalp hair specimen. The dotted horizontal line at 1.0 represents the reference control level of each trace element.

### 2.5. Infantile Time Window in Neurodevelopment and for Therapy

The age at final diagnosis of autism spectrum disorders ranges from 3 to 6 years, although most cases of autism are diagnosed by the age of three and as early as 14 months [34]. In facts, zinc deficiency was detected in many of the infantile patients in the first 3 years of life (Figure 1), and high toxic metal burdens were also detected in the autistic subjects, especially in the younger children (Table 2). Thus, for treatment/prevention of autism spectrum disorders, its early screening and estimation is necessary and it is desirable to early check any metabolic and/or mineral disorders for the infants and children with autistic symptoms, though there are serious limitations of diagnosis of the younger children. It should be considered that the pathogenesis of neurodevelopment disorders might start in prenatal phase and be progressive within the time window for diagnosis.

### 2.6. Autism-Related Genes and Epigenetic Alteration by Mineral Disorders

Zinc is a structural component of zinc-finger proteins and a transcriptional regulator, and influences some candidate genes reported to be associated with the development of autism, such as MTF1 (metal-responsive transcription factor 1), metallothionein, ZnT5 (zinc transporter 5), COMMD1 (COMM domain-containing protein 1), ERK1 (extracellular signal-regulated kinase 1), TrkB (tyrosine-related kinase B), and ProSAP/Shank (proline-rich synapse-associated protein/SH and multiple ankyrin repeat domains) that themselves are involved in zinc signalling and homeostasis [35,36,37,38,39,40]. Thus, zinc deficiency observed in the autistic subjects (Figure 1) might induce critical epigenetic alterations to provide a central mechanism of gene/environment interaction to interfere with neuronal maturation during early development [32,38,39].

In addition, high toxic metal burdens detected in the autistic patients (Table 2) might contribute to the mechanism of gene/environment interaction, because cadmium and arsenic have been reported to be candidate factors that induce epigenetic alterations [15,16,17,18,19] and neurodevelopmental disorders [20].

## 3. Discussion

In this metallomics study of human scalp hair concentrations of 26 trace elements for 1,967 children with autistic disorders aged 0–15 years, we demonstrate that many of the patients, especially in the infants aged 0–3 year-old, are suffering from marginal to severe zinc- and magnesium-deficiency and/or high burdens of several toxic metals such as aluminium, cadmium and lead.

Zinc is well-accepted as essential trace element that plays important roles in nucleic acid/protein synthesis, cell replication, tissue growth and repair, especially in pregnant women and infants. In fact, zinc ions function as the active centers in more than 300 kinds of enzymes, and about 10% in the total gene-coded proteins have been known to have zinc-finger sequences [38,39,40,41,42], emphasizing the physiological importance of this trace element. In brain, especially in the hippocampus, zinc is co-stored with glutamate in pre-synaptic vesicles in the excitatory neuron terminal, is released from them and controls the activity of excitatory glutamate receptors on the post-synaptic excitable membrane [43,44]. Thus, zinc deficiency is known associated with not only various pathological conditions, including dysgeusia, delayed wound healing, impaired immunity and retarded growth, but also neurodegenerative diseases and neurodevelopment disorders [45,46,47,48,49].

Recently we reported that many infants with autistic disorders are suffering from marginal to severe zinc deficiency, suggesting considerable relationship of infantile zinc deficiency with autism [32]. Furthermore, we have determined scalp hair concentrations of 26 trace elements for 1,967 subjects with autism spectrum disorders and demonstrated that infantile autistic children are liable to deficiency in magnesium and calcium next to zinc, but not in the other essential metals (Table 1) [33]. These findings suggest that autistic infants and probably infants generally have a characteristic liability to zinc- and magnesium-deficiency, because larger amounts of the essential metals (per kg body weight) are needed for the development and growth.

There are numerous studies with the same theme reporting nutritional status and mineral deficiencies in autistic children [50,51,52,53,54]. However, the conclusions of their studies, in which the restricted age (over 4-years-old) of children and number of minerals were examined, were not consistent, and the critical environmental factors remained to be established. In our metallomics analysis study for the 1,967 autistic children aged 0–15 years-old, we were able to demonstrate not only the critical and environmental epigenetic factor (zinc- and magnesium-deficiency and high burdens of aluminium, cadmium, lead and so on) but also the presence of another critical factor, “infantile window” in neurodevelopment and probably for therapy [32,33].

Recently, Gebremedhin *et al*. [55] reported that compared to pregnant women aged 15–24 years, those aged 25–34 and 35–49 years had 1.57 (95% CL: 1.04–2.34) and 2.18 (95% CL: 1.25–3.63) times higher risk of zinc deficiency, respectively. Their study may demonstrate that old age pregnancy is negatively associated to zinc status, maybe suggesting that one of the origins of the high incidence rate of infantile zinc deficiency may be higher age pregnancy of their mothers. Recently, Kurita *et al*. [56] reported that zinc deficiency in utero induces foetal epigenetic alterations of histone modifications in metallothionein 2 promoter region having metal responsive elements in 1-day-old and 5-week-old mice, of which pregnant mother were fed low zinc diet from gestation day 8 until delivery.

Arnold *et al*. [57] reported that mean serum zinc level in children was significantly lower in attention-deficit/hyperactivity disorder (ADHD) group, and that serum zinc level correlated inversely with parent- and teacher-rated inattention in ADHD children. Furthermore, zinc treatment was reported significantly superior to placebo in reducing symptoms of hyperactivity, impulsivity and impaired socialization in ADHD patients [58,59]. Another preliminary human study showed that many children with ADHD have lower zinc concentration in comparison to healthy children and zinc supplement as an adjunct to methylphenidate has favourable effects in the treatment of ADHD children, pointing to the possible association of zinc deficiency and ADHD pathophysiology [60].

Kozielec *et al*. [61] have reported that in 116 hyperactive children with ADHD, magnesium deficiency was found in 95% of the subjects, most frequently in hair (77.6%), next in red-blood cells (58.6%) and in blood serum (33.6%). Furthermore, they reported that in the group of ADHD children given 6 months of magnesium supplementation, a significant decrease of hyperactivity and increase in hair magnesium contents has been achieved [62]. Mousain-Bosc *et al*. [63] also reported that 52 hyper-excitable children have low intra-erythrocyte magnesium levels with normal serum magnesium values, and that magnesium/vitamin B6 supplementation can restore the erythrocyte magnesium levels to normal and improve their abnormal behaviours. They also reported that thirty-three children with clinical symptoms of pervasive developmental disorder or autism (PDD) exhibit significantly lower red blood cell magnesium values, and that the combination therapy with magnesium/vitamin B6 for 6 months improved significantly PDD symptoms in 23/33 children (*p* < 0.0001) with concomitant increases in intra-erythrocyte magnesium values [64].

Recently, Ochi *et al*. [65] found that hair magnesium concentration, but not its serum level, was significantly (*p* < 0.01) inversely-associated with left ventricular hypertrophy in hemodialysis patients, suggesting that hair magnesium concentration is a useful intracellular biomarker independent of its serum level. In a preliminary metallomics study for healthy volunteers, we have observed a high significant correlation between whole blood levels and scalp hair levels of trace elements, but little relation between their serum levels and whole blood levels (unpublished observation). These findings suggest that as biomarker specimen representing mineral dynamics in human body, whole blood/erythrocyte and hair samples are superior to extra-cellular fluids such as serum or plasma for metallomics analysis, although it is necessary to consider that there is a problem of contamination of some trace-elements due to artificial hair treatment such as permanent and colouring.

Recently, dietary restriction-induced zinc deficiency has been reported to up-regulate intestinal zinc-importer (ZIP4) and induce the increase in ZIP4 protein located to the plasma membrane of enterocytes [66,67]. This adoptive response to zinc deficiency is known to lead to increasing in the risk of high-uptake of toxic metals such as cadmium and lead [68]. Thus, infants with zinc deficiency are liable to increased risk of absorbing high amount of toxic metals and retaining them in their bodies, as shown in Figure 4, which demonstrates a high significant inverse relationship between zinc and lead level. These findings suggest that the increased toxic metal burdens concomitant with zinc deficiency may also epigenetically contribute to the pathogenesis of this disorder.

Deficiency in magnesium/calcium seems further enhance the toxic effects of lead (Pb) on cognitive and behavioural development in children [69]. A significant inverse relationship between dietary calcium intake and blood lead concentrations was found in 3,000 American children examined as part of NHANES II [69]. Elevated blood lead levels are found in some children diagnosed with autism and are associated with the development of ADHD [70,71].

About 250,000 children in the U.S.A. were reported to have high blood lead (Pb) levels over the current Level of Concern of 10 µg/dL [72], despite significant progresses over the past half century in reducing child lead poisoning rates [73]. Therefore, the U.S. Centers for Disease Control and Prevention (CDC) has lowered the Level of Concern from 10 µg/dL to 5 µg/dL [74]. This major change in national policy is based on a large and growing body of evidence showing that even single-digit blood Pb levels have significant impacts on Intelligence Quotients, risk for Attention Deficit Hyperactivity Disorder (ADHD), cardiovascular disease, and kidney function [75,76,77,78,79].

The most common lead exposure pathway for children are ingestion or inhalation of lead-bearing road dusts, whether in the household or outdoor environment [80,81,82], and its most common sources are fossil fuels, asphalt and paints (lead chromate or lead carbonate) [82,83,84]. In addition, maternal cigarette smoking has been reported to be associated with lower zinc and higher cadmium and lead concentrations in their neonates [85,86]. These toxic metals accumulated in the maternal bone tissues are co-transferred with calcium to foetal and new-born bodies through activated bone-resorption during pregnancy and lactation [85,86,87,88]. In fact, a recent birth cohort study for new-borns in Nepal shows that the motor cluster score was inversely associated with the cord blood levels of lead and arsenic, suggesting that high exposures to Pb and As during the prenatal period may induce retardation during in-utero neurodevelopment [89].

For mercury and arsenic, the maximum burden levels of 9.3- and 33.5-fold of the reference level (Table 2) may also epigenetically play a pathogenic role in the respective autistic individuals, even though their incidence rates were 2.8% or less. It remains to be established that these mineral disorders induce the epigenetic deficits in autism-related candidate genes. In near future, we hope it will be clarified what type of metallomics profiles is associated with what disorder in various behaviour/neurological deficits in autism spectrum disorders.

It is demonstrated that many autistic infants are suffering from marginal to severe zinc- and magnesium-deficiency and/or high toxic metal burdens of aluminium, cadmium, lead and so on. These findings suggest that infantile autistic patients with mineral disorders may respond to a novel evidence-based nutritional approach which supplements deficient nutrients and detoxifies accumulated toxic metals. This evidence-based nutritional approach may yield a new vista into early screening/assessment and treatment/prevention of infantile patients with autism spectrum disorders including the suspects. Well-controlled intervention studies for this novel nutritional therapy are desired to establish the epigenetic roles of infantile mineral imbalances in the pathogenesis of neurodevelopment disorders and to develop an early screening and therapy of neurodevelopment disorders such as autism spectrum disorders, ADHD and learning disorder.

## 4. Conclusions

This overview demonstrates that many of infantile patients with autism spectrum disorders suffer from marginal to severe zinc- and magnesium-deficiency and/or high toxic metal burdens, and that these mineral disorders (mineral imbalances) in bodies may play principal, epigenetic roles as environment factors in the pathogenesis of the neurodevelopment disorders. In addition, it is suggested that there is a critical time window “infantile window” in neurodevelopment and probably for treatment and prevention of these disorders. In near future, an introduction of innovative clinical tests such as metabolomics and metallomics analysis is desired for early estimation and treatment of neurodevelopment disorders.
